# Reproductive isolation due to prezygotic isolation and postzygotic cytoplasmic incompatibility in parasitoid wasps

**DOI:** 10.1002/ece3.5588

**Published:** 2019-08-20

**Authors:** Kerstin König, Petra Zundel, Elena Krimmer, Christian König, Marie Pollmann, Yuval Gottlieb, Johannes L. M. Steidle

**Affiliations:** ^1^ Fg Tierökologie Universitat Hohenheim Stuttgart Germany; ^2^ Gymnasium Renningen Renningen Germany; ^3^ Department of Animal Ecology and Tropical Biology Julius‐Maximilians‐Universitat Wurzburg Fakultat fur Biologie University of Würzburg Wurzburg Germany; ^4^ Robert H. Smith Faculty of Agriculture, Food and Environment Koret School of Veterinary Medicine Hebrew University of Jerusalem Rehovot Israel

**Keywords:** cytoplasmic incompatibility, endosymbiotic bacteria, *Lariophagus distinguendus*, parasitoid wasps, sexual isolation, speciation

## Abstract

The reproductive barriers that prevent gene flow between closely related species are a major topic in evolutionary research. Insect clades with parasitoid lifestyle are among the most species‐rich insects and new species are constantly described, indicating that speciation occurs frequently in this group. However, there are only very few studies on speciation in parasitoids. We studied reproductive barriers in two lineages of *Lariophagus distinguendus* (Chalcidoidea: Hymenoptera), a parasitoid wasp of pest beetle larvae that occur in human environments. One of the two lineages occurs in households preferably attacking larvae of the drugstore beetle *Stegobium paniceum* (“DB‐lineage”), the other in grain stores with larvae of the granary weevil *Sitophilus granarius* as main host (“GW‐lineage”). Between two populations of the DB‐lineage, we identified slight sexual isolation as intraspecific barrier. Between populations from both lineages, we found almost complete sexual isolation caused by female mate choice, and postzygotic isolation, which is partially caused by cytoplasmic incompatibility induced by so far undescribed endosymbionts which are not *Wolbachia* or *Cardinium*. Because separation between the two lineages is almost complete, they should be considered as separate species according to the biological species concept. This demonstrates that cryptic species within parasitoid Hymenoptera also occur in Central Europe in close contact to humans.

## Graphical abstract

We studied reproductive barriers in populations of two lineages of the parasitoid *Lariophagus distinguendus*, which prefer different hosts and occur in different habitats associated with humans. Between the lineages, there is almost complete sexual isolation caused by female mate choice, and postzygotic isolation, which is partially caused by cytoplasmic incompatibility induced by endosymbionts, which are neither Wolbachia nor Cardinium. Because separation is almost complete, the two lineages should be considered separate species according to the biological species concept.

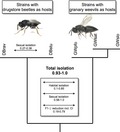

## 1 INTRODUCTION

To understand the process of speciation, it is essential to understand the evolution of isolating barriers which prevent gene flow between populations and eventually lead to the origin of new, reproductively isolated species according to the biological species concept (Butlin et al., 2012; Coyne & Orr, 2004; Sobel, Chen, Watt, & Schemske, 2010). Studies from many decades demonstrate that speciation can be the result of different processes. Depending on context, it has been categorized based on geography (allopatric vs. sympatric; Mayr, 1947), whether it occurs in divergent or similar ecological environments (ecological vs. mutation‐order; Schluter, 2009), or in the presence or the absence of (ecological) selection (Langerhans & Riesch, 2013; Sobel et al., 2010). An additional division is provided by Seehausen et al. (2014) who distinguish a fast and a slow route of speciation. The fast route starts with divergent ecological or sexual selection causing the quick evolution of extrinsic postzygotic and prezygotic barriers, while slowly evolving intrinsic postzygotic barriers only arise later. The slow route starts with the emergence of intrinsic barriers, which are then followed by extrinsic postzygotic and prezygotic barriers. Thus, ecological and sexual barriers as well as intrinsic postzygotic barriers seem to be most important for the first steps of divergence during speciation (Seehausen et al., 2014).

Divergence due to ecological factors is termed ecological speciation (Rundle & Nosil, 2005; Schluter, 2009; Sobel et al., 2010). Thereby, extrinsic selection caused by ecological factors like resources, climate, habitat, or other organisms results in the emergence of (a) prezygotic isolation, for example, because organisms live in different habitats, or (b) extrinsic postzygotic isolation due to reduced viability of migrants and hybrids in a foreign niche (Seehausen et al., 2014).

Sexual isolation can result in speciation when mate preferences and mating signals diverge between two populations and lead to communication breakdown and therefore to prezygotic isolation between the populations (Panhuis, Butlin, Zuk, & Tregenza, 2001). The importance of sexual selection for speciation is underlined by the observation that a divergence of sexual signals is often associated with the speciation process (Barraclough, Harvey, & Nee, 1995; Gray & Cade, 2000; Mendelson, Martin, & Flaxman, 2014; Panhuis et al., 2001; Wyatt, 2014). This divergence might be caused by the interaction between sexes and is therefore independent of ecology, but often is due to interactions with the environment. Therefore, it is considered to be part of ecological speciation (Rundle & Nosil, 2005; Ritchie, 2007; Schluter, 2009; Sobel et al., 2010; Rundle & Rowe, 2018; but also see Maan & Seehausen, 2011).

The third important component of speciation, intrinsic postzygotic isolation is mainly due to incompatibilities between genomes of divergent populations, termed Dobzhansky–Muller incompatibilities (DM or DMI) or Bateson–Dobzhansky–Muller incompatibilities (BDM) which lead to infertility, inviability, or other negative fitness effects in hybrids (Coyne & Orr, 2004; Lowry, Modliszewski, Wright, Wu, & Willis, 2008; Presgraves, 2010). A special case of postzygotic isolation is cytoplasmic incompatibility (CI) induced by endosymbiotic bacteria (Brucker & Bordenstein, 2012). These bacteria prevent the production of offspring when infected males are mated to uninfected females, by causing defects in the first mitotic division of the zygote (Serbus, Casper‐Lindley, Landmann, & Sullivan, 2008; Tram, Fredrick, Werren, & Sullivan, 2006). These endosymbionts are transmitted vertically to the offspring only by the females and have no effect when females and males are both infected, or when only females are infected. Therefore, CI increases the frequency of the inducing endosymbionts in the host population, by supporting infected females at the expense of noninfected females (Werren, 1997; Werren, Baldo, & Clark, 2008). So far, only two bacteria, *Wolbachia* and *Cardinium*, have been described to induce CI (White, 2011).

Hymenopterous parasitoids are one of the most species‐rich groups in the animal kingdom, with about 50,000 described species (Godfray, 1994). Numerous species are still unknown, especially in the tropics (Gokhman, 2018), and it is believed that hymenopterous parasitoids might comprise about 20% of all insects, or between 530,000 and 6,000,000 species (Forbes, Bagley, Beer, Hippee, & Widmayer, 2018; LaSalle & Gauld, 1991). Thus, as stated by Askew (1968) for the largest parasitoid superfamily Chalcidoidea (Noyes, 2016), this group is “in a state of active evolution at the present time.” This makes them highly suitable to study speciation. Despite this fact, there are only few systematic studies on pre‐ and postzygotic barriers of parasitoid wasps, but many studies on molecular, behavioral, and ecological traits to identify cryptic species (Bredlau & Kester, 2015; Danci, Schaefer, Schopf, & Gries, 2006; Desneux et al., 2009; Gebiola, Kelly, Hammerstein, Giorgini, & Hunter, 2016; Gebiola, White, et al., 2016; Kaiser et al., 2015; Rincon, Bordat, Löhr, & Dupas, 2006). The most extensive study on pre‐ and postzygotic barriers exists for the genus *Nasonia* (Chalcidoidea: Pteromalidae; Beukeboom, Koevoets, Morales, Ferber, & Zande, 2015; Bordenstein, O'Hara, & Werren, 2001; Breeuwer & Werren, 1995; Clark, O'Hara, Chawla, & Werren, 2010; Giesbers et al., 2013; Koevoets, Niehuis, Zande, & Beukeboom, 2012; Niehuis et al., 2013). Isolation by CI caused by different *Wolbachia* strains is the most important barrier within this genus (Raychoudhury, Baldo, Oliveira, & Werren, 2009) and seems to have initiated separation at least between *N. longicornis* and *N. giraulti* (Bordenstein et al., 2001). In addition, allopatry and intrinsic postzygotic isolation affecting males were important as initial barriers (Breeuwer & Werren, 1995; Clark et al., 2010).

**Figure 1:**
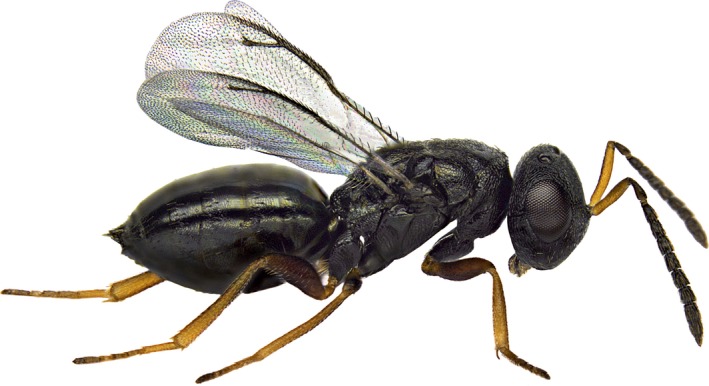
Male of *Lariophagus distinguendus*. Copyright SMNS (Johannes Reibnitz)

In the present study, we addressed reproductive barriers in the chalcidoid parasitoid *Lariophagus distinguendus* (Förster; Figure 1) with the aim to better understand speciation in parasitoid Hymenoptera. *L. distinguendus* is attacking beetle larvae living in grains, seeds, or cocoons (Niedermayer & Steidle, 2013; Steidle & Schöller, 1997). For many decades, its general biology, ecology, and use for the biological control of stored product pests have been extensively studied by many authors (reviewed by Niedermayer, Pollmann, & Steidle, 2016). König et al. (2015) reported on two distinct lineages of *L. distinguendus* which are ecologically separated. One prefers drugstore beetles *Stegobium paniceum* (L.) (Anobiidae) as hosts and is found in households on beetle‐infested products (“DB‐lineage”), while the other is mostly found in granaries attacking the granary weevil *Sitophilus granarius* (L.) (Dryophthoridae: Curculionoidea) (“GW‐lineage”). Because recent findings demonstrate that the DB‐lineage is ancestral (König et al., 2019), a host switch must have occurred, from drugstore beetles to granary weevils (König et al., 2015, 2019). Interestingly, the population GWpfo, which belongs to the GW‐lineage, maintained this ancestral preference for drugstore beetles but was collected from granary weevils in a grain store. Molecular analyses revealed considerable genetic differences between the two lineages (König et al., 2015), which also have different numbers of chromosomes (König et al., 2019). However, reproductive barriers between the lineages have not been studied and it is unclear if they represent conspecific ecotypes or separate species according to the biological species concept (Mayr, 1969).

We studied reproductive barriers in five populations, two from the DB‐lineage and three from the GW‐lineage, to distinguish between barriers which occur early during separation, that is within the lineages, and barriers which occur later, that is between the lineages (Coyne & Orr, 2004; Jennings, Snook, & Hoikkala, 2014). Potential sexual prezygotic isolation was addressed by studying courtship and mating behavior. Postzygotic isolation, that is, the absence of fertilization, was studied by counting the number of hybrid F_1_ female offspring. Only the number of F1 female offspring was used as a parameter, because males are haploid in Hymenoptera and emerge from eggs without fertilization. To address the role of endosymbionts causing CI as isolating barrier, we analyzed reproductive isolation between a single focal pair of populations (one of each lineage) after treatment with and without antibiotics.

The study revealed that both lineages are almost completely isolated and should be considered as separate species. We found one case of sexual isolation between two populations of the same species, which occur on the same host, indicating that speciation can be initiated by sexual isolation due to the divergence of sexual pheromones. Speciation is completed in this taxon by ecological separation, strong sexual isolation and by postzygotic barriers, including endosymbiont induced cytoplasmic incompatibility.

## 2 MATERIALS AND METHODS

### 2.1 Insects

The experiments were performed with wasps from five populations of two lineages of *L. distinguendus* collected by us or sent to us by colleagues. Wasps of the DB‐lineage were collected in pantries in private homes on drugstore beetles: DBrav, collected in 2008 in Ravensburg (Germany) and DBstu, collected in 2007 in Stuttgart (Germany). Wasps of the GW‐lineage were collected on granary weevils in grain stores: GWpfo collected in 2005 in Pforzheim (Germany), GWslo collected in 1996 in Slough (Great Britain), and GWsat collected in 2010 in Satrup (Germany). Thus, most populations (except of GWslo) were from the same geographic area.

A standard rearing technique was applied for all populations: 50–70 freshly emerged wasps were placed on host‐infested substrate and kept under constant rearing conditions of 26°C, 45% r.h. and a photoperiod of 16L:8D. Developmental time under these conditions is about 20 days in females and 19 days in males (Ryoo, Hong, & Yoo, 1991). Wasps of DBrav and DBstu were reared on 3rd to 4th instar larvae of the drugstore beetle *S. paniceum* infesting koi pellets (Hikari Friend, Kamihata Fish Industry Group, Kyorin Corporation, Japan) in glass jars (diameter 12 cm, 16 cm height) with a ventilated lid. Wasps of GWpfo, GWslo, and GWsat were reared on 3rd to 4th instar larvae of the granary weevil *S. granarius* infesting wheat grain (*Triticum aestivum* L.; cultivar: Batis; Saaten‐Union GmbH, Hannover, Germany) in Petri dishes. To exclude the potential impact of different rearing conditions in our experiments on the occurrence and number of F_1_ female hybrid offspring with DBrav and DBstu, we used wheat grains instead of KOI‐pellets as rearing substrate for the drugstore beetles. Apart from that, rearing conditions were as described above with the exception that 1 ml water was used to moisten grains in order to facilitate oviposition of female wasps.

To rear drugstore beetles, about 1 g of newly emerged unsexed adult drugstore beetles (about 700 beetles) was placed in a Petri dish on 80 g food pellets for koi fish or on 40 g wheat grain moistened with 1 ml water for oviposition and left there until they died. After six weeks, *L. distinguendus* were placed on the infested pellets or wheat grains, depending on the experiment. To rear granary weevils, unsexed adult granary weevils (2.7 g, about 600 beetles) were placed in jars (diameter 12 cm, 16 cm height) with a ventilated lid on 40 g of moistened wheat grain (40 ml H_2_O on 1 kg grain) for oviposition. After one week, adult beetles were removed. Beetle cultures were kept at 27°C and 65% r.h. and natural daylight conditions.

### 2.2 Behavioral studies

To study sexual isolation, experiments were performed similar to well‐established mating experiments with *Nasonia* as no‐choice experiments with single females and males (Giesbers et al., 2013). Individuals from all five populations in all cross combinations were tested under standardized conditions (23°C and 50%–60% r.h.). To ensure that females were unmated, wasps were separated directly after emergence from the cultures and kept in small groups of up to 10 individuals of the same sex in Petri dishes on a moist filter paper. After 48 hr, one female and one male wasp were placed in a mating arena consisting of a glass Petri dish (diameter 14 mm) closed with a glass plate (30 mm × 30 mm). Mating behavior in *L. distinguendus* consists of courtship behaviors “wing fanning” and “antennal stroking” by the males, “receptivity signal” by the female (lowering the head, folding down the antennae and exposing the genitalia), and finally copulation (Ruther, Homann, & Steidle, 2000). These behaviors were observed with a stereo‐microscope for a maximum observation time of 20 min and recorded using “The Observer v. 5.0” (Noldus). According to our experience, most pairs with females and males from the same population mate during this time. However, as demonstrated by the occurrence of female offspring (see below) in couples were no mating has been observed, some mating between females and males from different populations must have occurred after this observation time. Twenty pairs were studied per combination.

### 2.3 Hybrid F_1_ females

This experiment aimed to study reduced fertilization after mating of individuals from different populations, and/or reduced viability of hybrid F_1_ females. Each pair from the previous behavioral studies was placed in a small Petri dish with 20 wheat grains infested by 3rd and 4th instar larvae of either drugstore beetles (when females were from populations DBrav or DBstu) or granary weevils (when females were from populations GWpfo, GWslo, or GWsat). This enabled mating for those pairs, which did not mate in the behavioral experiment, as well as oviposition by females on their preferred hosts. After 48 hr, the adult wasps were discarded. After 30 days, the number and sex of emerging F_1_ offspring were registered. To study reduced fertilization as barrier between different populations, we used the occurrence of F_1_ offspring (Figure S1). To study reduced viability of hybrid F_1_ females, we used the mean number of female F_1_ offspring from each combination. Thereby, we based our analysis only on those pairs, which produced female offspring to exclude the influence of missing copulations.

### 2.4 Role of CI as postzygotic barrier

To address the role of endosymbionts causing CI as isolating barrier, we analyzed reproductive isolation between a single focal pair of populations (DBrav and GWpfo, i.e., one of each lineage) after treatment with and without antibiotics. To establish endosymbiont free lines, freshly emerged wasps were mated and then placed into a Petri dish with a filter paper soaked with a tetracycline‐sugar solution (1 mg/ml tetracycline in 10% sucrose; Breeuwer & Werren, 1993) for 24 hr. Then, wasps were placed on their breeding host as described above. This procedure was repeated for seven generations. For the experiments, females and males of the F29 to F42 generation after termination of antibiotic treatment were used. The absence of endosymbionts in these wasps was verified by PCR (Table 3).

For the experiments, wasps were collected immediately after emergence to avoid mating and kept in same‐sex groups as described above. One virgin female and one virgin male were placed together on either 5 g pellets infested by larvae of the drugstore beetle (when females were from DBrav) or 10 g wheat grains infested by larvae of the granary weevil (when females were from GWpfo) and kept under constant conditions of 26°C and 45% r.h. at cycle of 16L:8D hours for 16 days. The number and sex of emerging wasps were registered after 30 days.

### 2.5 Detection of CI‐inducing endosymbionts

Ethanol preserved wasps were washed with sterile double distilled water (ddW) and grinded thoroughly in 10 μl of lysis buffer (mixture: 5 μl Tris 1 M, 1 μl EDTA, 5 μl Igepal, 50 μl Proteinase K, 939 μl ddW) in a Petri dish covered with aluminum foil and parafilm to extract their DNA. Additional lysis buffer (30 μl) was added and the whole fluid was pulled up and down with a pipette once. The lysed wasp was then transferred to a 1.5 ml tube and incubated for 15 min at 65°C and 10 min at 95°C. DNA concentration and nucleic acid/protein ratio (260/280 nm) of the conducted DNA extractions were measured using a Thermo Scientific NanoDrop 2000 spectrophotometer. Samples with concentrations between 40 and 500 ng/μl and ratios above 1.8 were used for further analysis. Samples with lower concentration or poor quality were discarded.

Five to ten specimens of each of the five wasp populations and different generations of antibiotic‐treated strains of DBrav and GWpfo were tested for *Wolbachia* and *Cardinium* using polymerase chain reaction (PCR) amplification with several specific and with one universal bacterial 16SrRNA primer (Table 1). PCR conditions are given in Table 2. PCR amplifications were performed in 13 μl of reaction buffer containing 6.5 μl Promega GoTaq^®^ Green Master Mix 2X, 2 μl template DNA, 0.5 μl 10 μM of each primer, and 3.5 μl ddW using a Biometra professional Basic Thermocycler. Sterile, double distilled water was used as a negative control. Positive controls were provided from *Nesidiocoris tenuis* and *Culicoides* spp. Resulting PCR products were loaded on a 1.5% agarose gel stained with ethidium bromide for visualization under UV‐light. Molecular weight size markers used were Norgen LowRanger 100 bp DNA Ladder and New England BioLabs Inc. Quick‐Load^®^ Purple 2‐Log DNA Ladder.

**Table 1:** Primers used in this study

Primer	Specificity (target gene)	Sequence (5′→3′)	Reference
wsp81F	*Wolbachia* (wsp)	TGGTCCAATAAGTGATGAAGAAAC	Braig, Zhou, Dobson, and O'Neill (1998)
wsp691R		AAAAATTAAACGCTACTCCA	
Car‐sp‐F	*Cardinium*(16S rDNA)	CGGCTTATTAAGTCAGTTGTGAAATCCTAG	Nakamura et al. (2009)
Car‐sp‐R		TCCTTCCTCCCGCTTACACG	
CLOf	Cytophaga‐like‐organisms (CLO) (16S rDNA)	GCG GTG TAA AAT GAG CGT G	Weeks, Robert, and Richard (2003)
CLOr1		ACC TMT TCT TAA CTC AAG CCT	
gyrFWD	*Cardinium* (gyraseB subunit)	TTG CTC CGG ACC ATT CTA TC	Nakamura et al. (2009)
gyrRVS		GTT TCT ACC GCT CCT TGC AC	
27F	Universal (16S rRNA)	AGA GTT TGA TCC TGG CTC AG	Weisburg, Barns, Pelletier, and Lane (1991)
1949R		CTA CGG CTA CCT TGT TAC GA	

**Table 2:** Primer pairings and PCR conditions used in this study

Primer	PCR conditions	Amplicon length (bp)
Wsp set	95°C 2 min, 35 cycles 92°C 30 s/58°C 30 s/72°C 30 s, 72°C 5 min	610
Car‐sp‐set	95°C 1 min, 35 cycles 95°C 30 s/57°C 30 s/72°C 1 min, 72°C 5 min	544
CLO set	94°C 4 min, 35 cycles of 94°C 40 s/57°C 40 s/72°C 45 s, 72°C 5 min	450
gyrB set	95°C 5 min, 35 cycles of 95°C 30/60°C 30 s/72°C 1 min, 72°C 10 min	500
Universal 16S rRNA	95°C 5 min, 35 cycles of 95°C 30/60°C 30 s/72°C 1 min, 72°C 10 min	1,450

### 2.6 Calculation of isolation indices

Reproductive isolation (RI) is the strength of any pre‐ or postzygotic barrier and represents an estimate how much gene flow is reduced by this barrier (Coyne & Orr, 2004). Based on Sobel and Chen (2014) RI varies from −1 (the barrier allows for gene flow only between heterospecifics) over 0 (gene flow is random) to 1 (only gene flow between conspecifics). RI was calculated according to the following formula:

$$1-2*\frac{\left(H\right)}{\left(H\right)+\left(C\right)}$$

*H* refers to events, which enable gene flow between heterospecifics (i.e., frequency of mating between heterospecifics or the number of offspring after mating of heterospecifics), while *C* refers to events, which enable conspecific gene flow.

RI for habitat isolation and immigrant inviability was calculated according to Nosil, Vines, and Funk (2005), using data from König et al. (2015). For habitat isolation, we used the host preference data (frequency of drilling in the original and the foreign host), for immigrant inviability we used the fecundity data (offspring number with original and foreign host). To calculate RI for sexual isolation, we used the data of the mating experiments presented here. To calculate RI for postzygotic isolation, we used the mean number of female F_1_ offspring.

Total isolation (*T*) varies between 0 (no isolation) and 1 (full isolation between populations). It was calculated as the sum of reproductive isolation caused by all different barriers during the life history of the wasps, starting with the prezygotic barriers habitat isolation, immigrant inviability, and sexual isolation, and ending up with postzygotic isolation, that is the number of F1 ♀ offspring. For each barrier, we used the absolute contribution (AC). It considers this part of gene flow that has not already been prevented by previous stages of reproductive isolation (Ramsey, Bradshaw, & Schemske, 2003). It was calculated as follows:

$$\text{AC}\;\text{for}\;\text{the}\;\text{first}\;\text{barrier}:\;{\text{AC}}_{1}={\text{RI}}_{1}$$

$$\text{AC}\;\text{for}\;\text{the}\;\text{second}\;\text{barrier}:\;{\text{AC}}_{2}={\text{RI}}_{2}*\left(1-{\text{AC}}_{1}\right).$$

$$\text{AC}\;\text{for}\;\text{the}\;\text{third}\;\text{barrier}:\;{\text{AC}}_{3}={\text{RI}}_{3}*\left[1-\left({\text{AC}}_{1}+{\text{AC}}_{2}\right)\right].$$

Total isolation (*T*) was calculated using the following formula:

$$T=\sum_{i=1}^{m}{\text{AC}}_{i}.$$

### 2.7 Estimation of evolutionary divergence over sequence pairs between groups

The number of base differences per site from averaging over all sequence pairs between groups was estimated using the partial mitochondrial cytochrome oxidase I (COI) sequences with a total of 679 positions in the final dataset. This analysis involved 10 nucleotide sequences (GenBank Accession numbers: KJ867375–KJ867378 and KJ867383–KJ867388) from König et al. (2015). Evolutionary analyses were conducted in MEGA X (Kumar, Stecher, Li, Knyaz, & Tamura, 2018). Additional sequences from *Nasonia* (EU746609–EU746612) from Oliveira, Raychoudhury, Lavrov, and Werren (2008) were used to compare evolutionary divergence.

### 2.8 Statistical analysis

Data from behavioral studies and data on the occurrence of F1 female offspring were analyzed using R (R Core Team, 2016). In an overall comparison, the occurrence of the different behaviors or the presence of F1‐ females was compared for all combinations using a 2 × 5 Fisher exact test or 2 × 4 Fisher exact test, respectively. In case of a significant overall difference, single combinations were compared using a 2 × 2 Fisher exact test, followed by sequential Bonferroni correction. The number of the F_1_ female hybrids were compared with R (R Core Team, 2016) using a linear mixed model (Bates, Mächler, Bolker, & Walker, 2014) with female population as random factor followed by ANOVA (Fox & Weisberg, 2011) and the Tukey tests (Hothorn, Bretz, & Westfall, 2008).

## 3 RESULTS

### 3.1 Behavioral studies

**Figure 2:**
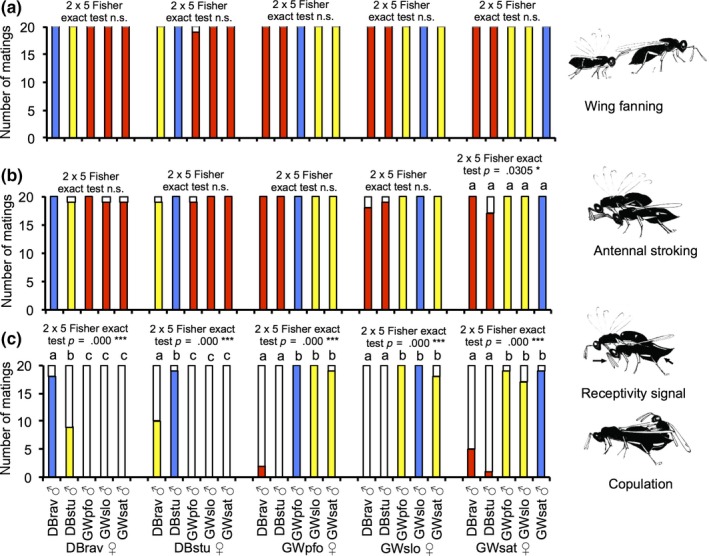
Occurrence of courtship and copulation in pairs consisting of females and males from five populations of *Lariophagus distinguendus*. (a) Wing fanning; (b) antennal stroking and head nodding; (c) receptivity signal by the female and copulation. Colored parts of the bars refer to pairs in which the specific behavior was observed; white parts of bars indicate pairs for which the specific behavior was not observed; blue bars: female and male from the same population and the same lineage; yellow bars: females and males from different populations, but from the same lineage; red bars: females and males from different populations and different lineages. For the females of each population we compared the occurrence of each behavior in experiments with males from the different populations using the 5 × 2 Fisher exact test followed by the Bonferroni corrected 2 × 2 Fisher exact test for single comparisons. Bars with different lower case letters are statistically significant at *p *<.05; n.s. = not significant. For each combination 20 pairs were tested

The mating experiments revealed that males reacted equally well to females of all populations from both lineages, while females were much more restricted in their response. All experiments were grouped according to the female population. For every group, we compared the occurrence of each behavior. Experiments with males and females from the same population were used as control and compared to the other combinations. “Wing fanning” toward females and “antennal stroking” on the females was performed by males in almost all replicates of the intralineage and interlineage combinations. There were no significant differences (Figure 2; Tables S1–S3). In contrast, the receptivity signal by females and subsequent copulation were observed almost exclusively in the intralineage combinations (blue and yellow bars in Figure 2) and only rarely in the interlineage combinations (red bars in Figure 2). For all tested groups, there were significant differences between intralineage and interlineage combinations (Figure 2; Tables S4 and S5). Interestingly, a significant (about 50%) decrease in the occurrence of receptivity signal and copulations was also observed between the two populations DBrav and DBstu, which belong to the same lineage and occurred on the same host.

### 3.2 Postzygotic barriers causing hybrid female inviability

**Figure 3:**
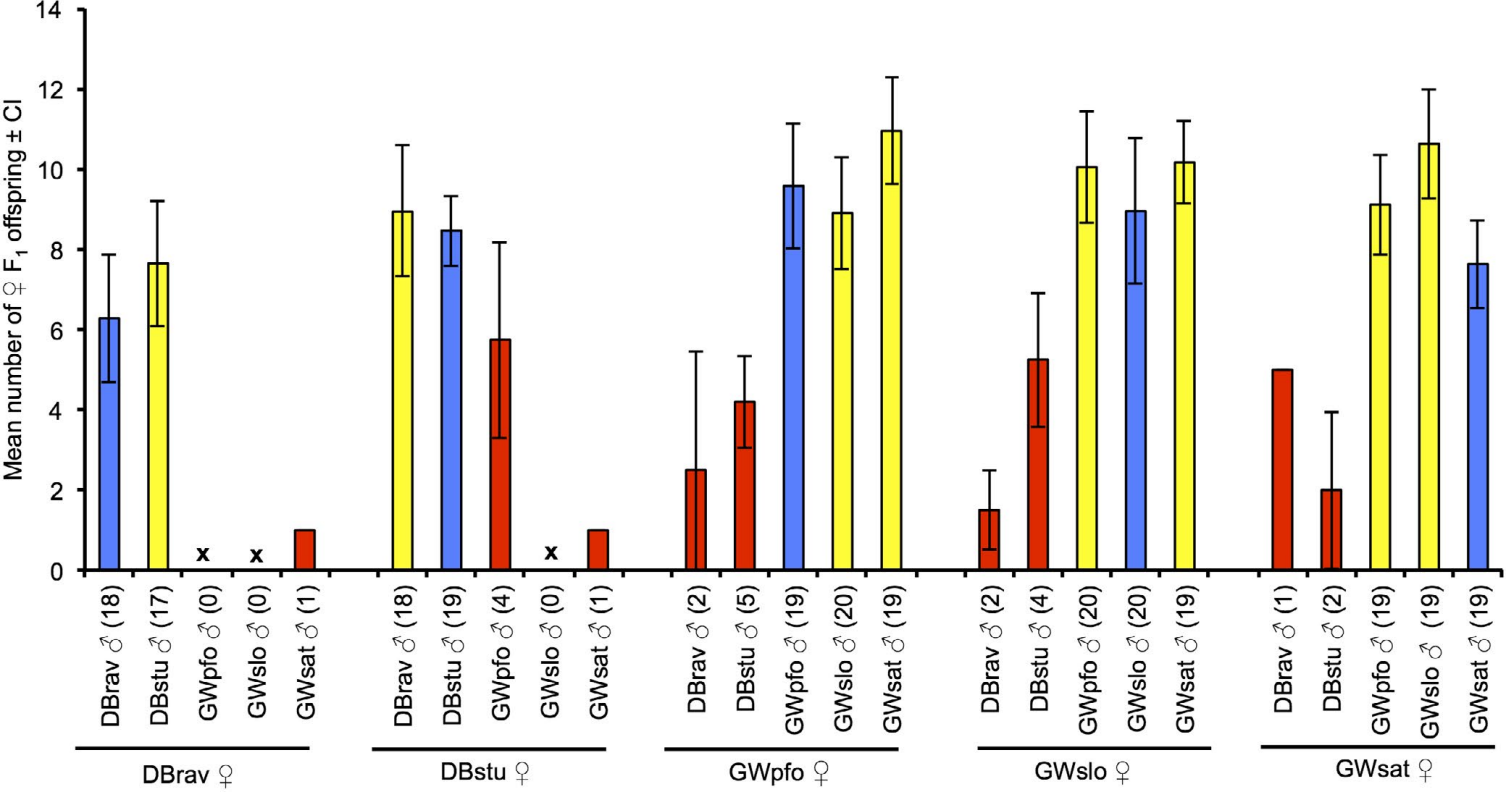
Mean number of F_1_ female offspring (+ confidence intervals) from pairs consisting of females and males from five populations of *Lariophagus distinguendus*. Blue bars: female and male from the same population and the same lineage; yellow bars: females and males from different populations, but from the same lineage; red bars: females and males from different populations and different lineages; *x* = no F_1_ female offspring available for testing. The number of pairs tested for each combination is given in brackets. Differences in offspring numbers between pairs from the same population, pairs from different populations, but from the same lineage and pairs from different lineages were analyzed using a linear mixed model with female population as random factor followed by ANOVA and Tukey tests

In Figure 3 and Figure S1 experiments are grouped according to the female population as in the behavioral experiments. While most pairs from the nonhybrid control crosses with females and males from the same population (number of replicates for blue bars in Figure 3; blue bars in Figure S1; Table S6) or from populations of the same lineage (number of replicates for yellow bars in Figure 3; yellow bars in Figure S1; Table S6) produced female offspring, this was not the case with pairs from the interlineage combinations (number of replicates for red bars in Figure 3; red bars in Figure S1; Table S6). Thereby, female offspring occurred in several pairs that did not mate in the behavioral experiments. Obviously, mating must have taken place in these pairs in the Petri dish with hosts after the behavioral experiment was finished.

It is unclear, if missing female offspring was caused by the absence of copulations with foreign males, a prezygotic barrier, by missing fertilization as a result of postmating–prezygotic isolation, or by postzygotic isolation (Coyne & Orr, 2004). Therefore, to analyze the number of F1‐female offspring that were produced by nonhybrid and hybrid parental pairs, we studied only the pairs with female offspring, as indication that copulation and fertilization has taken place. Due to the low number of these pairs in the combinations between lineages, data were pooled for statistical analysis. All data from control combinations (female and male from the same population, blue bars in Figure 3; *n* = 95), the hybrid intralineage combinations (female and male from different population but from the same lineage, yellow bars in Figure 3; *n* = 151), and the interlineage combinations (female and male from different lineages; red bars in Figure 3; *n* = 22) were compared using a linear mixed model with female population as random factor followed by ANOVA and the Tukey tests. This revealed a significant difference within the three groups (ANOVA for groups: *χ* ^2^ = 72.00, *df* = 2, *p* ≤ .001; Figure 3). Single comparisons showed that hybrid crosses between lineages (see red bars in Figure 3) produced significantly less female offspring than hybrid crosses within a lineage (yellow bars in Figure 3; Tukey test: *z* = 8.412, *p *≤ .001) and nonhybrid controls (blue bars in Figure 3; Tukey test: *t* = ‒6.463, *p* ≤ .001). Hybrid intralineage crosses produced more hybrid F_1_ female offspring than nonhybrid controls (Tukey test: *z* = 2.984, *p* ≤ .0083).

**Figure 4:**
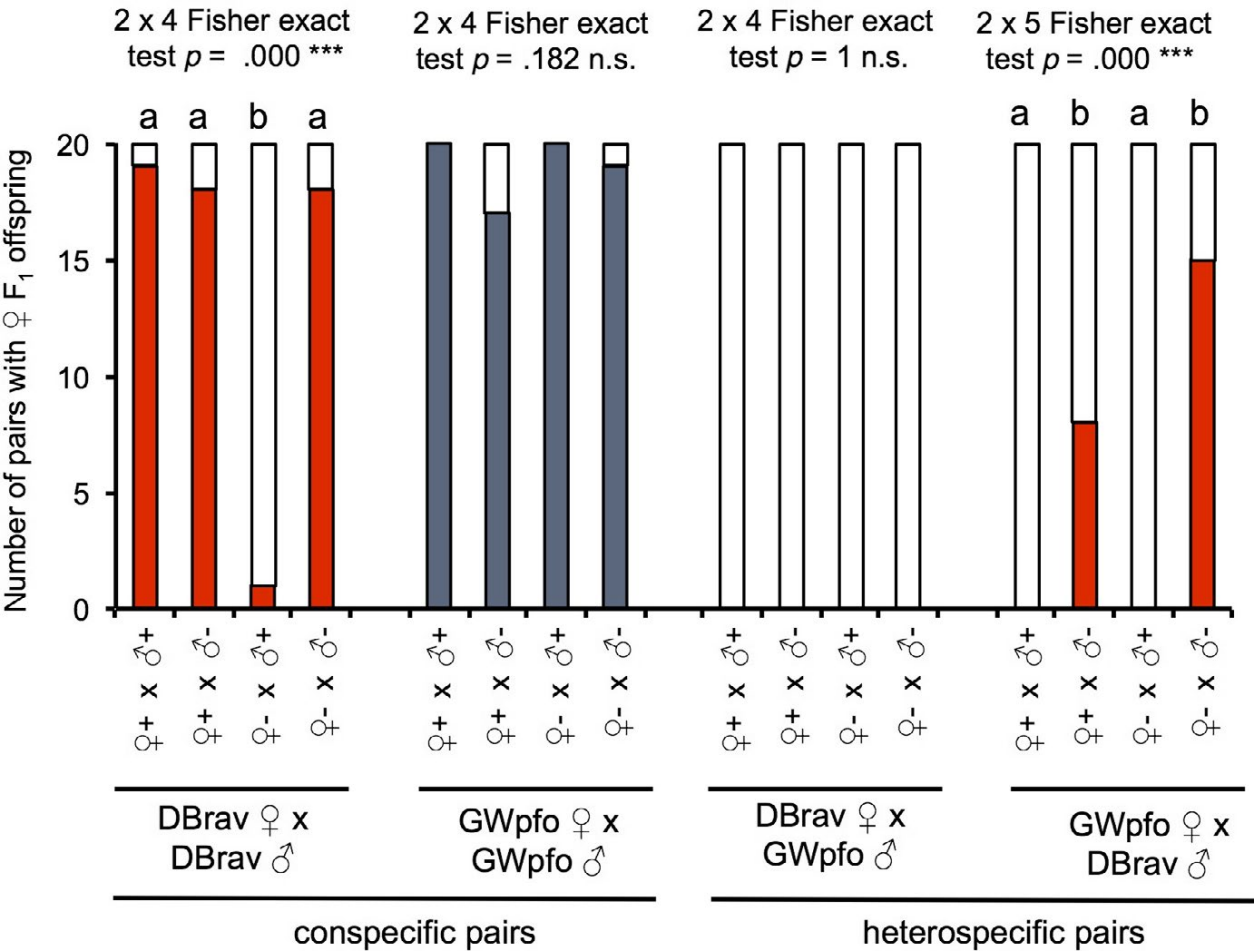
Occurrence of F_1_ female offspring from conspecific and heterospecific pairs of the populations DBrav and GWpfo of *Lariophagus distinguendus*. Individuals were carrying putative endosymbionts (♀+, ♂+) or were endosymbiont free due to antibiotic treatment (♀−, ♂−). Red and blue parts of the bars refer to pairs in which F_1_ female offspring emerged; white parts of bars indicate pairs, which did not produce female F_1_ offspring; Bars within one group with different lower case letters are significantly different at *p* < .05 (Bonferroni corrected *χ* ^2^ test). n.s. = not significant. For each combination 20 pairs were tested

Cytoplasmic incompatibility (CI) was studied with wasps from the DBrav and the GWpfo populations, which were untreated or treated with antibiotic. PCR‐analyses revealed that wasps treated with antibiotic were free of endosymbionts (Table 3). In experiments with the DBrav population, significantly less of the endosymbiont‐free females produced F1 female offspring when mated with endosymbiont‐carrying males, as compared to all the other combinations with this population (Figure 4; Tables S7 and S8). In contrast, all combinations of the GWpfo population had female offspring, regardless of the presence of putative endosymbionts (Figure 4; Table S7). When GWpfo‐females were mated with endosymbiont free DBrav males, they produced significantly more F_1_ female offspring as compared to the other combinations with endosymbiont carrying DBrav males (Figure 4; Tables S7 and S8). This indicates that the DB‐lineage, but not the GW‐lineage was infected with CI‐inducing bacteria. Diagnostic PCR for *Wolbachia* and *Cardinium* revealed that the DBrav population, as well as the population DBstu from the same lineage, and antibiotic‐treated wasps were not infected with either bacteria, while GWpfo and the two other populations from this lineage (GWslo, GWsat) were infected with *Wolbachia* (all tested wasps were positive). Positive controls and negative controls resulted as expected (Data not shown). Thus, CI‐inducing bacteria in the DB‐lineage must be different from *Wolbachia* or *Cardinium*. Data from the crosses DBrav♀ × GWpfo♂ did not reveal F_1_ female offspring in all treatment combinations, indicating that additional barriers were involved, such as sexual isolation (see Section 4).

**Table 3:** Presence of the endosymbiotic bacteria *Wolbachia* and *Cardinium* in wasps from different populations of *L. distinguendus* based on PCR analysis

Population	wsp (*Wolbachia*)	16S drDNA (*Cardinium*)	Universal 16S rRNA
DBrav (*n* = 10)	0/10^a^	0/10	10/10
DBstu (*n* = 10)	0/10	0/10	10/10
GWpfo (*n* = 10)	10/0	0/10	10/10
GWslo (*n* = 10)	10/10	0/10	10/10
GWsat	10/10	n.a.	10/10
DBrav AB^b^ F38^c^ (*n* = 5)	0/5	0/5	0/5
DBrav AB F39 (*n* = 5)	0/5	0/5	0/5
DBrav AB F42 (*n* = 10)	0/10	0/10	0/5
GWpfo AB F38 (*n* = 5)	0/5	0/5	0/5
GWpfo AB F39 (*n* = 5)	0/5	0/5	0/10
GWpfo AB F42 (*n* = 10)	0/10	0/10	0/10
GWslo AB F29 (*n* = 5)	0/5	0/5	0/5
GWslo AB F30 (*n* = 10)	0/10	0/10	0/10

a Number of specimen with endosymbionts/number of endosymbiont free specimen.

b AB indicates that wasps strain has been treated with antibiotic.

c F indicates number of generations after end of antibiotic treatment.

### 3.3 Isolation indices

Isolation indices (RI) for the different barriers revealed only slight isolation due to sexual isolation between DBrav × DBstu within the DB‐lineage, and no isolation within the GW‐lineage (Table 4). Sexual isolation between the lineages was almost complete. Isolation due to habitat choice and immigrant inviability (calculated using data from König et al., 2015) only occurred between the two lineages and ranged from absent to strong. Postzygotic isolation due to a reduced number of F_1_ females from hybrid pairs varied from weak to absolute. Total isolation based on all barriers together was complete or almost complete between the lineages.

**Table 4:** Uncorrected p‐distances as measure for evolutionary divergence between populations of *Lariophagus distinguendus*, isolation indices for different barriers calculated according to Sobel and Chen (2014), and total isolation using the absolute contribution of each barrier according to Ramsey et al. (2003)

Cross combination (♀/♂)	Uncorrected p‐distances (COI)	Habitat isolation	Isolation due to immigrant inviability	Sexual isolation	Isolation due to reduced number F_1_ ♀ offspring	*T* (Total isolation)
DBstu/DBrav	0.000	0	0	0.267	−0.027	0.247
DBrav/DBstu		0	0	0.385	−0.098	0.325
GWsat/GWslo	0.004	0	0	0.056	−0.164	−0.099
GWslo/GWsat		0	0	0.053	−0.063	−0.007
GWslo/GWpfo	0.022	0	0	0	−0.058	−0.058
GWpfo/GWslo		0	0	0	0.037	0.247
GWsat/GWpfo	0.024	0	0	0	−0.088	0.084
GWpfo/GWsat		0	0	0.026	−0.067	−0.039
GWsat/DBstu	0.138	0.855	n.st.	0.900	0.585	0.994
DBstu/GWsat		0.729	0.763	1	0.789	1
GWsat/DBrav	0.138	0.855	n.st.^2^	0.583	0.208	0.952
DBrav/GWsat		0.687	0.548	1	0.725	1
GWpfo/DBrav	0.140	0.092	−0.084	0.818	0.586	0.926
DBrav/GWpfo		0.687	0.548	1	1	1
GWpfo/DBstu	0.140	0.092	−0.084	1	0.390	1
DBstu/GWpfo		0.729	0.763	1	0.191	1
GWslo/DBstu	0.141	0.538	0.220	1	0.261	1
DBstu/GWslo		0.729	0.763	1	1	1
GWslo/DBrav	0.141	0.538	0.220	1	0.713	1
DBrav/GWslo		0.687	0.548	1	1	1

Note

Habitat isolation and immigrant viability are based on the data on host preference and fecundity from König et al. (2015).

## 4 DISCUSSION

We comparatively studied pre‐ and postzygotic reproductive barriers in five populations of the parasitoid wasp *L. distinguendus*, which belong to two distinct lineages and might well be different species. In the following, we will discuss ecological, sexual and intrinsic postzygotic barriers that have been identified between the populations.

### 4.1 Ecological isolation

Because the two lineages of *L. distinguendus* have been found on two different host species (DB‐lineage on larvae of drugstore beetles, GW‐lineage on larvae of granary weevils) and in different environments (households vs. granaries, respectively), they are ecologically isolated. We calculated isolation indices for habitat isolation and immigrant viability as suggested by Nosil et al. (2005) based on the host recognition behavior on grains infested by the two different host and the offspring number on the two hosts in an earlier study (König et al., 2015). Isolation between the two lineages is not absolute, but varies between the different combinations of populations from zero (0.09 for habitat isolation and ‒0.08 for isolation due to immigrant inviability), to strong (0.86 and 0.76, respectively). This is for two reasons. First, populations of the GW‐lineage have a high fecundity on both hosts. Second, the population GWpfo has an innate preference for drugstore beetles, although it was found on granary weevils as hosts and genetically belongs to the GW‐lineage (König et al., 2015). Therefore, we assumed that during the process of divergence a host switch occurred from drugstore beetles to granary weevils, and that the GWpfo population has retained an ancestral innate preference for drugstore beetles which is also found in the populations of the DB‐lineage (König et al., 2015). The assumption that the DB‐lineage is ancestral was supported by our recent karyological study (König et al., 2019).

### 4.2 Sexual isolation

Our behavioral studies revealed that females signaled receptivity and mated only when courted by males from the same lineage. In addition, we observed a significant 50% decrease in reaction of females from the DB‐lineage to males from the other population of the same lineage. The absence of a receptivity signal by the female upon courtship by heterospecific males was most likely due to the male oral gland pheromone, which is applied onto the female antenna during courtship (König et al., 2015). Sealing the male mandibles with glue to block the pheromone release resulted in the same behavior observed in our experiments, that is missing female receptivity (König et al., 2015). Obviously, the male oral pheromone differs between the two species and also between the two populations within the DB‐species. Isolation indices for sexual isolation based on these behavioral data are close to zero between the three populations from the GW‐lineage, at around 0.3 between the two populations from the DB‐lineage, and close to 1 between populations from different lineages. Thus, female mate choice results in slight sexual isolation between the two populations of the DB‐lineage and in almost complete sexual isolation between the two lineages.

The isolation between the two DB‐populations is remarkable, because both prefer the same host (König et al., 2015), there were no molecular differences between the two populations based on mitochondrial and nuclear markers (König et al., 2015), and no other barriers are known so far. This demonstrates that the separation of populations during speciation in *L. distinguendus* can start with sexual isolation based on female mate choice. However, the fact that no sexual isolation was detected between populations of the GW‐lineage indicates that this finding cannot be generalized for our study system.

In contrast to the response of the females, almost all males reacted to heterospecific females by courtship behavior, despite the fact that female cuticular hydrocarbons that stimulate the male response (Ruther et al., 2000) differ between the two species (Kühbandner, Hacker, Niedermayer, Steidle, & Ruther, 2012). This sex‐specific difference in the reaction to varying male and female pheromones is in agreement with the asymmetric tracking hypothesis (Phelan, 1992; Wyatt, 2014). It states that due to differences in paternal investment and therefore a different number of potential offspring, males are expected to react to a larger variation in sexual signals as compared to the females, which should be more choosy to avoid offspring with unsuitable males with potentially reduced fitness (Phelan, 1992, 1997b, 1997a).

### 4.3 Postzygotic isolation and CI

Hybrid female inviability as intrinsic postzygotic barrier was studied based on the occurrence and the mean number of F_1_ female offspring in mixed pairs from different populations and lineages. Only few mixed pairs from different lineages produced female offspring (Figure S1) and if so the female offspring number was significantly reduced. In contrast, mixed pairs of the same lineage had even more female offspring than control pairs, most likely due to heterosis (Benvenuto et al., 2012). Isolation indices were almost 0 between populations from the same lineage and ranged from 0.19 to 1 between populations from different lineages.

Potential reasons for the reduction in female offspring from pairs of different lineages are (a) sexual isolation, (b) gametic isolation, a postmating‐prezygotic barrier, (c) postzygotic barriers like CI induced by endosymbionts, (d) chromosomal rearrangements, or (e) genic incompatibilities (Coyne & Orr, 2004). Sexual isolation may have caused the absence of female offspring, but it cannot explain the reduced number of female offspring because means from these data were calculated only from pairs where copulation has taken place, as demonstrated by the presence of at least one daughter. The role of gametic isolation is very difficult to study in *L. distinguendus*, which often lays its single eggs in the powdery feces of the host where they cannot be retrieved for further examination. We studied the presence of bacterial induced CI between DBrav (representative for the DB‐lineage) and GWpfo (for the GW‐lineage). These studies revealed that DBrav, but not GWpfo, is infected with bacteria, which induce CI between DBrav and GWpfo. This explains part of the observed postzygotic isolation between the two lineages. Remarkably, we were not able to find any evidence for the presence of *Wolbachia* or *Cardinium* in both populations of the DB‐lineage. These are the only two bacteria for which CI has been described (White, 2011). Obviously, the DB‐lineage is carrying an hitherto unknown, CI‐inducing bacterium. We are currently working on its identification.

CI cannot explain the reduction of female offspring in pairs consisting of GWpfo‐males and DBrav‐females because males from GWpfo do not cause CI, regardless whether they had been treated with antibiotics or not. Based on our findings on sexual isolation, we consider it most likely that this reduction is due to female mate choice, that is the rejection of GWpfo‐males by DBrav‐females. However, at least parts of the effect might be also caused by the fact that the two *L. distinguendus* species have different chromosome numbers (Gokhman, 2015), or by genetic incompatibilities. More studies are required to answer this question.

### 4.4 Isolating barriers in other parasitoid systems

Isolating barriers for *L. distinguendus* agree with barriers in other parasitoid systems. Prezygotic ecological isolation and sexual isolation seem to be common (Bredlau & Kester, 2015; Danci et al., 2006; Gounou, Chabi‐Olaye, Poehling, & Schulthess, 2008; Heimpel, Antolin, Franqui, & Strand, 1997; Joyce et al., 2010; Kaiser et al., 2015; Sundaralingam, Hower, & Kim, 2001), also in populations which must have been separated only very recently (Desneux et al., 2009). CI due to the endosymbionts *Wolbachia* or *Cardinium* has been reported in several parasitoid systems (*Wolbachia*: e.g., Bordenstein et al., 2001; Bordenstein & Werren, 2007; Branca, Ru, Vavre, Silvain, & Dupas, 2011; Gebiola, White, et al., 2016; *Cardinium*: Gotoh, Noda, & Ito, 2007; Perlman, Kelly, & Hunter, 2008; Zhang, Zhao, & Hong, 2012; Gebiola, Kelly, et al., 2016; Gebiola, Giorgini, et al., 2017). Apart from CI, only very few studies exist on intrinsic postzygotic barriers, especially for those barriers affecting males. Male infertility and inviability in hybrids of closely related species have been reported for the two parasitoid species *N. longicornis* and *N. giraulti* (Bordenstein et al., 2001; Bordenstein & Werren, 2007; Clark et al., 2010). For the *L. distinguendus* system we are currently studying these barriers.

### 4.5 Two species of *L. distinguendus*

Total isolation indices revealed that reproductive isolation is complete or almost complete between the two lineages. The uncorrected p‐distances of 0.138 to 0.141 based on COI between the *L. distinguendus* lineages are mostly higher than the distances reported for other parasitoids which are considered separate species, for example 0.065 between *N. giraulti* and *N. longicornis* and 0.089 between *N. giraulti*/*N. longicornis* and *N. vitripennis* (both calculated based on data from Oliveira et al., 2008), and 0.034 to 0.144 for *Encarsia* species (Gebiola, Monti, et al., 2017). For the *Nasonia*‐combinations, divergence times of 0.4–0.51 MYA for the split between *N. giraulti* and *N. longicornis* (Campbell, Steffen‐Campbell, & Werren, 1994; Raychoudhury et al., 2009), and 1 MY between *N. giraulti*/*N. longicornis* and *N. vitripennis* were estimated (Werren & Loehlin, 2009). Thus, the two *Lariophagus* lineages must be considered true, separate species according to the biological species concept (Mayr, 1969) representing another example of cryptic diversity within parasitoid Hymenoptera. This supports the idea that within the Hymenoptera a large number of species are still undescribed, even in Europe with a seemingly well studied fauna (Aguiar et al., 2013).

## 5 CONCLUSIONS

Our study revealed that *L. distinguendus*, a parasitoid which occurs in human environments and has been studied for a long time (Niedermayer et al., 2016), consists of at least two different species. These species are reproductively separated by (a) ecological isolation, (b) almost absolute sexual isolation due to female mate choice, (c) postzygotic isolation due to CI caused by a yet undescribed endosymbiotic bacterium, (d) and other, hitherto unknown barriers. Interestingly, slight sexual isolation, based on the divergence of sexual pheromones was found between two populations of one species, which are not ecologically separated. This indicates that sexual isolation has the potential to initiate speciation. However, because this finding is restricted to only one conspecific population pair of *L. distinguendus*, it cannot be generalized. Therefore it is unclear, if speciation in *L. distinguendus* started with prezygotic isolation based on ecological or sexual selection, or by postzygotic isolation due to CI. More studies with closely related populations of *L. distinguendus* are required to answer this question.

## CONFLICT OF INTEREST

None declared.

## AUTHOR CONTRIBUTION

Kerstin König conceived parts of the study, performed some of the experiments and wrote parts of the ms, Petra Zundel performed some of the experiments, Elena Krimmer and Marie Pollmann performed some of the experiments and conducted parts of the molecular analyses, Christian König analyzed the molecular data on the phylogenetic relationships of the wasps, Yuval Gottlieb conceived and supervised the molecular analyses of the endosymbionts and wrote parts of the ms, Johannes Steidle conceived the study and wrote parts of the manuscript.

## Data Availability

Original data are deposited in Dryad https://doi.org/10.5061/dryad.p19fp25.
