# Supplementary material for: Reproductive isolation due to prezygotic isolation and postzygotic cytoplasmic incompatibility in parasitoid wasps
Source: Ecol Evol. 2019 Aug 20;9(18):10694–706. doi: 10.1002/ece3.5588 (PMC6787869; doi:10.1002/ece3.5588)
Supplement: Supplementary file 1 [file ECE3-9-10694-s001.docx]

Supporting information

**Table S1:** Test statistics (*p*-values) for data in Fig. 1 on the occurrence of fanning by males towards females in pairs consisting of females and males from five populations of *Lariophagus distinguendus*. Twenty pairs were tested per combination. An overall comparison was performed for all combinations in which females were from the same population using the 5 x 2-Fisher exact test. n.s.: not significant.

| Combination 1 | Combination 2 | Combination 3 | Combination 4 | Combination 5 | *p*-value |  |
| --- | --- | --- | --- | --- | --- | --- |
| DBrav♀DBrav♂ | DBrav♀DBstu♂ | DBrav♀GWpfo♂ | DBrav♀GWslo | DBrav♀GWsat♂ | 1 | n.s. |
| DBstu♀DBrav♂ | DBstu♀DBstu♂ | DBstu♀GWpfo♂ | DBstu♀GWslo♂ | DBstu♀GWsat♂ | 1 | n.s. |
| GWpfo♀DBrav♂ | GWpfo♀DBstu♂ | GWpfo♀GWpfo♂ | GWpfo♀GWslo♂ | GWpfo♀GWsat♂ | 1 | n.s. |
| GWslo♀DBrav♂ | GWslo♀DBstu♂ | GWslo♀GWpfo♂ | GWslo♀GWslo♂ | GWslo♀GWsat♂ | 1 | n.s. |
| GWsat♀DBrav♂ | GWsat♀DBstu♂ | GWsat♀GWpfo♂ | GWsat♀GWslo♂ | GWsat♀GWsat♂ | 1 | n.s. |

**Table S2:** Test statistics (*p*-values) for data in Fig. 1 on the occurrence of antennal stroking of males towards females in pairs consisting of females and males from five populations of *Lariophagus distinguendus*. Twenty pairs were tested per combination. An overall comparison was performed for all combinations in which females were from the same population using the 5 x 2-Fisher exact test. n.s.: not significant; * *p* <= 0.05.

| Combination 1 | Combination 2 | Combination 3 | Combination 4 | Combination 5 | *p*-value |  |
| --- | --- | --- | --- | --- | --- | --- |
| DBrav♀DBrav♂ | DBrav♀DBstu♂ | DBrav♀GWpfo♂ | DBrav♀GWslo | DBrav♀GWsat♂ | 1 | n.s. |
| DBstu♀DBrav♂ | DBstu♀DBstu♂ | DBstu♀GWpfo♂ | DBstu♀GWslo♂ | DBstu♀GWsat♂ | 1 | n.s. |
| GWpfo♀DBrav♂ | GWpfo♀DBstu♂ | GWpfo♀GWpfo♂ | GWpfo♀GWslo♂ | GWpfo♀GWsat♂ | 1 | n.s. |
| GWslo♀DBrav♂ | GWslo♀DBstu♂ | GWslo♀GWpfo♂ | GWslo♀GWslo♂ | GWslo♀GWsat♂ | 0.505 | n.s. |
| GWsat♀DBrav♂ | GWsat♀DBstu♂ | GWsat♀GWpfo♂ | GWsat♀GWslo♂ | GWsat♀GWsat♂ | 0.03525 | * |

**Table S3:** Test statistics (*p*-values) for data in Fig. 1 on the occurrence of courtship behaviour of males towards females in pairs consisting of females and males from five populations of *Lariophagus distinguendus*. Twenty pairs were tested per combination. The presence or absence of the receptivity signal by females was compared between combinations using the 2 x 2 Fisher exact test. Because significant overall differences in the 5 x 2 Fisher exact Test (s. Table S2) were only found in the combinations with females from the GWsat population, only these combinations were analysed.

| Combination 1 | Combination 2 | p-value | significance after sequential Bonferroni-correction |
| --- | --- | --- | --- |
| GWsat♀DBrav♂ | GWsat♀DBstu♂ | 0.427 | n.s. |
| GWsat♀DBrav♂ | GWsat♀GWpfo♂ | 1.000 | n.s. |
| GWsat♀DBrav♂ | GWsat♀GWslo♂ | 1.000 | n.s. |
| GWsat♀DBrav♂ | GWsat♀GWsat♂ | 1.000 | n.s. |
| GWsat♀DBstu♂ | GWsat♀GWpfo♂ | 0.427 | n.s. |
| GWsat♀DBstu♂ | GWsat♀GWslo♂ | 0.427 | n.s. |
| GWsat♀DBstu♂ | GWsat♀GWsat♂ | 0.427 | n.s. |
| GWsat♀GWpfo♂ | GWsat♀GWslo♂ | 1.000 | n.s. |
| GWsat♀GWpfo♂ | GWsat♀GWsat♂ | 1.000 | n.s. |
| GWsat♀GWslo♂ | GWsat♀GWsat♂ | 1.000 | n.s. |

**Table S4:** Test statistics (*p*-values) for data in Fig. 1 on the occurrence of the receptivity signal by females and copulation behaviour in pairs consisting of females and males from five populations of *Lariophagus distinguendus*. Twenty pairs were tested per combination. An overall comparison was performed for all combinations in which females were from the same population using the 5 x 2-Fisher exact test. ****p* <= 0.001.

| Combination 1 | Combination 2 | Combination 3 | Combination 4 | Combination 5 | *p*-value |  |
| --- | --- | --- | --- | --- | --- | --- |
| DBrav♀DBrav♂ | DBrav♀DBstu♂ | DBrav♀GWpfo♂ | DBrav♀GWslo | DBrav♀GWsat♂ | 0.000 | *** |
| DBstu♀DBrav♂ | DBstu♀DBstu♂ | DBstu♀GWpfo♂ | DBstu♀GWslo♂ | DBstu♀GWsat♂ | 0.000 | *** |
| GWpfo♀DBrav♂ | GWpfo♀DBstu♂ | GWpfo♀GWpfo♂ | GWpfo♀GWslo♂ | GWpfo♀GWsat♂ | 0.000 | *** |
| GWslo♀DBrav♂ | GWslo♀DBstu♂ | GWslo♀GWpfo♂ | GWslo♀GWslo♂ | GWslo♀GWsat♂ | 0.000 | *** |
| GWsat♀DBrav♂ | GWsat♀DBstu♂ | GWsat♀GWpfo♂ | GWsat♀GWslo♂ | GWsat♀GWsat♂ | 0.000 | *** |

**Table S5:** Test statistics (*p*-values) for data in Fig. 1 on the occurrence of the receptivity signal by females and the occurrence of copulations in pairs consisting of females and males from five populations of *Lariophagus distinguendus*. Twenty pairs were tested per combination. The presence or absence of the receptivity signal by females was compared between combinations using the 2 x 2- Fisher exact test. n.s. not significant; **p* <= 0.01; ****p* <= 0.001.

| Combination 1 | Combination 2 | *p*-value | significance after sequential  Bonferroni-correction | |
| --- | --- | --- | --- | --- |
| DBrav♀DBrav♂ | DBrav♀DBstu♂ | 0.002 | * | |
| DBrav♀DBrav♂ | DBrav♀GWpfo♂ | 0.000 | *** | |
| DBrav♀DBrav♂ | DBrav♀GWslo♂ | 0.000 | *** | |
| DBrav♀DBrav♂ | DBrav♀GWsat♂ | 0.000 | *** | |
| DBrav♀DBstu♂ | DBrav♀GWpfo♂ | 0.004 | *** | |
| DBrav♀DBstu♂ | DBrav♀GWslo♂ | 0.004 | * | |
| DBrav♀DBstu♂ | DBrav♀GWsat♂ | 0.004 | * | |
| DBrav♀GWpfo♂ | DBrav♀GWslo♂ | 1.000 | | n.s. |
| DBrav♀GWpfo♂ | DBrav♀GWsat♂ | 1.000 | | n.s. |
| DBrav♀GWslo♂ | DBrav♀GWsat♂ | 1.000 | | n.s. |
| DBstu♀DBrav♂ | DBstu♀DBstu♂ | 0.008 | | * |
| DBstu♀DBrav♂ | DBstu♀GWpfo♂ | 0.000 | | *** |
| DBstu♀DBrav♂ | DBstu♀GWslo♂ | 0.000 | | *** |
| DBstu♀DBrav♂ | DBstu♀GWsat♂ | 0.000 | | *** |
| DBstu♀DBstu♂ | DBstu♀GWpfo♂ | 0.000 | | *** |
| DBstu♀DBstu♂ | DBstu♀GWslo♂ | 0.000 | | *** |
| DBstu♀DBstu♂ | DBstu♀GWsat♂ | 0.000 | | *** |
| DBstu♀GWpfo♂ | DBstu♀GWslo♂ | 1.000 | | n.s. |
| DBstu♀GWpfo♂ | DBstu♀GWsat♂ | 1.000 | | n.s. |
| DBstu♀GWslo♂ | DBstu♀GWsat♂ | 1.000 | | n.s. |
| GWpfo♀DBrav♂ | GWpfo♀DBstu♂ | 0.487 | | n.s. |
| GWpfo♀DBrav♂ | GWpfo♀GWpfo♂ | 0.000 | | *** |
| GWpfo♀DBrav♂ | GWpfo♀GWslo♂ | 0.000 | | *** |
| GWpfo♀DBrav♂ | GWpfo♀GWsat♂ | 0.000 | | *** |
| GWpfo♀DBstu♂ | GWpfo♀GWpfo♂ | 0.000 | | *** |
| GWpfo♀DBstu♂ | GWpfo♀GWslo♂ | 0.000 | | *** |
| GWpfo♀DBstu♂ | GWpfo♀GWsat♂ | 0.000 | | *** |
| GWpfo♀GWpfo♂ | GWpfo♀GWslo♂ | 1.000 | | n.s. |
| GWpfo♀GWpfo♂ | GWpfo♀GWsat♂ | 1.000 | | n.s. |
| GWpfo♀GWslo♂ | GWpfo♀GWsat♂ | 1.000 | | n.s. |
| GWslo♀DBrav♂ | GWslo♀DBstu♂ | 1.000 | | n.s. |
| GWslo♀DBrav♂ | GWslo♀GWpfo♂ | 0.000 | | *** |
| GWslo♀DBrav♂ | GWslo♀GWslo♂ | 0.000 | | *** |
| GWslo♀DBrav♂ | GWslo♀GWsat♂ | 0.000 | | *** |
| GWslo♀DBstu♂ | GWslo♀GWpfo♂ | 0.000 | | *** |
| GWslo♀DBstu♂ | GWslo♀GWslo♂ | 0.000 | | *** |
| GWslo♀DBstu♂ | GWslo♀GWsat♂ | 0.000 | | *** |
| GWslo♀GWpfo♂ | GWslo♀GWslo♂ | 1.000 | | n.s. |
| GWslo♀GWpfo♂ | GWslo♀GWsat♂ | 0.487 | | n.s. |
| GWslo♀GWslo♂ | GWslo♀GWsat♂ | 0.487 | | n.s. |
| GWsat♀DBrav♂ | GWsat♀DBstu♂ | 0.182 | | n.s. |
| GWsat♀DBrav♂ | GWsat♀GWpfo♂ | 0.000 | | *** |
| GWsat♀DBrav♂ | GWsat♀GWslo♂ | 0.000 | | *** |
| GWsat♀DBrav♂ | GWsat♀GWsat♂ | 0.000 | | *** |
| GWsat♀DBstu♂ | GWsat♀GWpfo♂ | 0.000 | | *** |
| GWsat♀DBstu♂ | GWsat♀GWslo♂ | 0.000 | | *** |
| GWsat♀DBstu♂ | GWsat♀GWsat♂ | 0.000 | | *** |
| GWsat♀GWpfo♂ | GWsat♀GWslo♂ | 0.605 | | n.s. |
| GWsat♀GWpfo♂ | GWsat♀GWsat♂ | 1.000 | | n.s. |
| GWsat♀GWslo♂ | GWsat♀GWsat♂ | 0.605 | | n.s. |


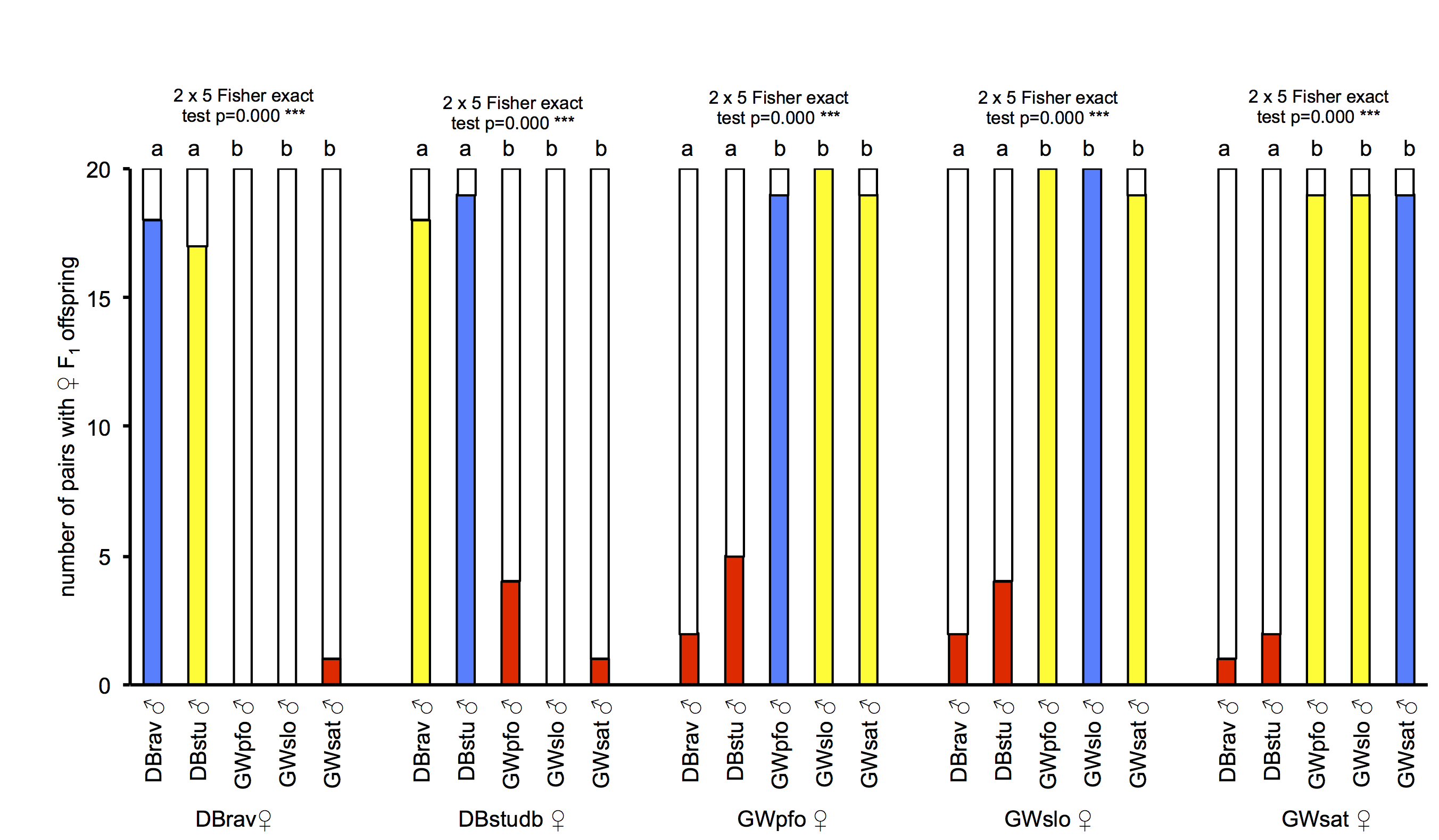


**Figure S1** Occurrence of F_1_ female offspring from pairs consisting of females and males from five populations of *Lariophagus distinguendus*. Coloured parts of the bars refer to pairs which produced F1-female offspring; white parts of bars indicate pairs with no F1-female offspring; Green bars: female and male from the same population and the same lineage; yellow bars: females and males from different populations, but from the same lineage; red bars: females and males from different populations and different lineages. For the females of each population we compared the occurrence of F1-female offspring in experiments with males from the different populations using the 5 × 2 Fisher exact test followed by the Bonferroni corrected 2 × 2 Fisher exact test for single comparisons. Bars with different lower case letters are statistically significant at *p*<=  0.05; n.s. not significant; ****p* <= 0.001. For each combination 20 pairs were tested.

**Table S6:** Test statistics (*p*-values) for data in Fig. S1 on the occurrence of F1-female offspring from pairs consisting of females and males from five populations of *Lariophagus distinguendus*. Twenty pairs were tested per combination. The presence or absence of F1-female offspring was compared between combinations using the 2 x 2- Fisher exact test. n.s. not significant;s ****p* < = 0.001.

| Combination 1 | Combination 2 | *p*-value | significance after sequential  Bonferroni-correction | |
| --- | --- | --- | --- | --- |
| DBrav♀DBrav♂ | DBrav♀DBstu♂ | 1.000 | n.s. | |
| DBrav♀DBrav♂ | DBrav♀GWpfo♂ | 0.000 | *** | |
| DBrav♀DBrav♂ | DBrav♀GWslo♂ | 0.000 | *** | |
| DBrav♀DBrav♂ | DBrav♀GWsat♂ | 0.000 | *** | |
| DBrav♀DBstu♂ | DBrav♀GWpfo♂ | 0.000 | *** | |
| DBrav♀DBstu♂ | DBrav♀GWslo♂ | 0.000 | *** | |
| DBrav♀DBstu♂ | DBrav♀GWsat♂ | 0.000 | *** | |
| DBrav♀GWpfo♂ | DBrav♀GWslo♂ | 1.000 | | n.s. |
| DBrav♀GWpfo♂ | DBrav♀GWsat♂ | 1.000 | | n.s. |
| DBrav♀GWslo♂ | DBrav♀GWsat♂ | 1.000 | | n.s. |
| DBstu♀DBrav♂ | DBstu♀DBstu♂ | 1.000 | | n.s. |
| DBstu♀DBrav♂ | DBstu♀GWpfo♂ | 0.000 | | *** |
| DBstu♀DBrav♂ | DBstu♀GWslo♂ | 0.000 | | *** |
| DBstu♀DBrav♂ | DBstu♀GWsat♂ | 0.000 | | *** |
| DBstu♀DBstu♂ | DBstu♀GWpfo♂ | 0.000 | | *** |
| DBstu♀DBstu♂ | DBstu♀GWslo♂ | 0.000 | | *** |
| DBstu♀DBstu♂ | DBstu♀GWsat♂ | 0.000 | | *** |
| DBstu♀GWpfo♂ | DBstu♀GWslo♂ | 0.106 | | n.s. |
| DBstu♀GWpfo♂ | DBstu♀GWsat♂ | 0.342 | | n.s. |
| DBstu♀GWslo♂ | DBstu♀GWsat♂ | 1.000 | | n.s. |
| GWpfo♀DBrav♂ | GWpfo♀DBstu♂ | 0.487 | | n.s. |
| GWpfo♀DBrav♂ | GWpfo♀GWpfo♂ | 0.000 | | *** |
| GWpfo♀DBrav♂ | GWpfo♀GWslo♂ | 0.000 | | *** |
| GWpfo♀DBrav♂ | GWpfo♀GWsat♂ | 0.000 | | *** |
| GWpfo♀DBstu♂ | GWpfo♀GWpfo♂ | 0.000 | | *** |
| GWpfo♀DBstu♂ | GWpfo♀GWslo♂ | 0.000 | | *** |
| GWpfo♀DBstu♂ | GWpfo♀GWsat♂ | 0.000 | | *** |
| GWpfo♀GWpfo♂ | GWpfo♀GWslo♂ | 1.000 | | n.s. |
| GWpfo♀GWpfo♂ | GWpfo♀GWsat♂ | 1.000 | | n.s. |
| GWpfo♀GWslo♂ | GWpfo♀GWsat♂ | 1.000 | | n.s. |
| GWslo♀DBrav♂ | GWslo♀DBstu♂ | 0.408 | | n.s. |
| GWslo♀DBrav♂ | GWslo♀GWpfo♂ | 0.000 | | *** |
| GWslo♀DBrav♂ | GWslo♀GWslo♂ | 0.000 | | *** |
| GWslo♀DBrav♂ | GWslo♀GWsat♂ | 0.000 | | *** |
| GWslo♀DBstu♂ | GWslo♀GWpfo♂ | 0.000 | | *** |
| GWslo♀DBstu♂ | GWslo♀GWslo♂ | 0.000 | | *** |
| GWslo♀DBstu♂ | GWslo♀GWsat♂ | 0.000 | | *** |
| GWslo♀GWpfo♂ | GWslo♀GWslo♂ | 1.000 | | n.s. |
| GWslo♀GWpfo♂ | GWslo♀GWsat♂ | 1.000 | | n.s. |
| GWslo♀GWslo♂ | GWslo♀GWsat♂ | 1.000 | | n.s. |
| GWsat♀DBrav♂ | GWsat♀DBstu♂ | 1.000 | | n.s. |
| GWsat♀DBrav♂ | GWsat♀GWpfo♂ | 0.000 | | *** |
| GWsat♀DBrav♂ | GWsat♀GWslo♂ | 0.000 | | *** |
| GWsat♀DBrav♂ | GWsat♀GWsat♂ | 0.000 | | *** |
| GWsat♀DBstu♂ | GWsat♀GWpfo♂ | 0.000 | | *** |
| GWsat♀DBstu♂ | GWsat♀GWslo♂ | 0.000 | | *** |
| GWsat♀DBstu♂ | GWsat♀GWsat♂ | 0.000 | | *** |
| GWsat♀GWpfo♂ | GWsat♀GWslo♂ | 1.000 | | n.s. |
| GWsat♀GWpfo♂ | GWsat♀GWsat♂ | 1.000 | | n.s. |
| GWsat♀GWslo♂ | GWsat♀GWsat♂ | 1.000 | | n.s. |

**Table S7:** Test statistics (*p*-values) for data in Fig. 3 on the occurrence of F1-female offspring from conspecific and heterospecific pairs of the populations DBrav and GWpfo of *Lariophagus distinguendus*. Individuals were carrying putative endosymbionts (♀+, ♂+) or were endosymbiont free due to antibiotic treatment (♀-, ♂-). An overall comparison was performed for all combinations with conspecific pairs from DBrav and GWpfo, and with heterospecific combinations with females from DBrav and GWpfo using the 4 x 2-Fisher exact test. ****p* <= 0.001.

| Combination 1 | Combination 2 | Combination 3 | Combination 4 | *p*-value |  |
| --- | --- | --- | --- | --- | --- |
| DBrav ♀+/♂+ | DBrav ♀+/♂- | DBrav ♀-/♂+ | DBrav ♀-/♂- | 0.000 | n.s. |
| GWpfo ♀+/♂+ | GWpfo ♀+/♂- | GWpfo ♀-/♂+ | GWpfo ♀-/♂- | 0.182 | *** |
| DBrav/GWpfo ♀+/♂+ | DBrav/GWpfo ♀+/♂- | DBrav/GWpfo ♀-/♂+ | DBrav/GWpfo ♀-/♂- | 1 | n.s. |
| GWpfo/DBrav ♀+/♂+ | GWpfo/DBrav ♀+/♂- | GWpfo/DBrav ♀-/♂+ | GWpfo/DBrav ♀-/♂- | 0.000 | *** |

**Table S8:** Test statistics (*p*-values) for data in Fig. 3 on the occurrence of F1-female offspring from conspecific pairs of the populations DBrav and heterospecific pairs of the populations DBrav and GWpfo of *Lariophagus distinguendus*. Individuals were carrying putative endosymbionts (♀+, ♂+) or were endosymbiont free due to antibiotic treatment (♀-, ♂-). Twenty pairs were tested per combination. The presence or absence of F1-female offspring was compared between combinations using the 2 x 2- Fisher exact test. n.s. not significant;s ****p* < = 0.001.

| Combination 1 | | Combination 2 | *p*-value | | | | significance after sequential  Bonferroni-correction | |
| --- | --- | --- | --- | --- | --- | --- | --- | --- |
| Dbrav ♀+/♂+ | Dbrav ♀+/♂- | | 1 | n.s. | | |  |  |
| Dbrav ♀+/♂+ | Dbrav ♀-/♂+ | | 0.000 | *** | | |  |  |
| Dbrav ♀+/♂+ | Dbrav ♀-/♂- | | 1 | n.s. | | |  |  |
| Dbrav ♀+/♂- | Dbrav ♀-/♂+ | | 0.000 | *** | | |  |  |
| Dbrav ♀+/♂- | Dbrav ♀-/♂- | | 1 | n.s. | | |  |  |
| Dbrav ♀-/♂+ | Dbrav ♀-/♂- | | 0.000 | *** | | |  |  |
| GWpfo/DBrav ♀+/♂+ | GWpfo/DBrav ♀+/♂- | | 0.003 | | *** | |  |  |
| GWpfo/DBrav ♀+/♂+ | GWpfo/DBrav ♀-/♂+ | | 1 | | n.s. | |  |  |
| GWpfo/DBrav ♀+/♂+ | GWpfo/DBrav ♀-/♂- | | 0.000 | | *** | |  |  |
| GWpfo/DBrav ♀+/♂- | GWpfo/DBrav ♀-/♂+ | | 0.003 | | *** | |  |  |
| GWpfo/DBrav ♀+/♂- | GWpfo/DBrav ♀-/♂- | | 0.054 | | n.s. | |  |  |
| GWpfo/DBrav ♀-/♂+ | GWpfo/DBrav ♀-/♂- | | 0.000 | | *** | |  |  |
